# Asymmetric Supercapacitors Using Porous Carbons and Iron Oxide Electrodes Derived from a Single Fe Metal-Organic Framework (MIL-100 (Fe))

**DOI:** 10.3390/nano13121824

**Published:** 2023-06-08

**Authors:** Seong Cheon Kim, Siyoung Q. Choi, Jeasung Park

**Affiliations:** 1 Department of Chemical and Biomolecular Engineering, Korea Advanced Institute of Science and Technology (KAIST), Daejeon 34141, Republic of Korea; grey_sckim@kaist.ac.kr (S.C.K.); sqchoi@kaist.ac.kr (S.Q.C.); 2 Korea Institute of Industrial Technology (KITECH), 89 Yangdaegiro-gil Ipjang-myeon Seobuk-gu, Cheonan-si 31056, Chungcheongnam-do, Republic of Korea; 3 KAIST Institute for the Nanocentury, Korea Advanced Institute of Science and Technology (KAIST), Daejeon 34141, Republic of Korea

**Keywords:** asymmetric supercapacitors (ASCs), hybrid type supercapacitor, MIL-100 (Fe), high performance, MOF-derived carbons (MDCs), α-Fe_2_O_3_ MOF-derived metal oxides (MDMO)

## Abstract

MOF-derived carbon (MDC) and metal oxide (MDMO) are superior materials for supercapacitor electrodes due to their high specific capacitances, which can be attributed to their high porosity, specific surface area (SSA), and pore volume. To improve the electrochemical performance, the environmentally friendly and industrially producible MIL-100 (Fe) was prepared using three different Fe sources through hydrothermal synthesis. MDC-A with micro- and mesopores and MDC-B with micropores were synthesized through carbonization and an HCl washing process, and MDMO (α-Fe_2_O_3_) was obtained by a simple sintering in air. The electrochemical properties in a three-electrode system using a 6 M KOH electrolyte were investigated. These novel MDC and MDMO were applied to an asymmetric supercapacitor (ASC) system to overcome the disadvantages of traditional supercapacitors, enhancing energy density, power density, and cyclic performance. High SSA materials (MDC-A nitrate and MDMO iron) were selected for negative and positive electrode material to fabricate ASC with KOH/PVP gel electrolyte. As-fabricated ASC resulted in high specific capacitance 127.4 Fg^−1^ at 0.1 Ag^−1^ and 48.0 Fg^−1^ at 3 Ag^−1^, respectively, and delivered superior energy density (25.5 Wh/kg) at a power density 60 W/kg. The charging/discharging cycling test was also conducted, indicating 90.1% stability after 5000 cycles. These results indicate that ASC with MDC and MDMO derived from MIL-100 (Fe) has promising potential in high-performance energy storage devices.

## 1. Introduction

Grave environmental consequences have resulted from the careless use of fossil fuels [1,2,3,4,5,6,7,8]. Concurrently, there has been an increase in energy demand as a result of the quick uptake of electric vehicles and the pervasiveness of portable electronics [5,6,8]. As a result, there is an urgent need for the development of high-efficiency energy storage technologies [1,2,3,4,7].

The two main candidates among the many energy storage technologies are batteries and supercapacitors. Lithium-ion batteries (LIBs), an example of an electrochemical battery, unquestionably have a higher energy density than their conventional equivalents. They cannot be used in high-power applications due to their comparatively low power density [5,6,9,10]. Supercapacitors, on the other hand, show greater potential due to their ability to give dependable, quick performance, increased power density, and improved durability. The low capacitance, however, is a significant barrier to their broad implementation [11,12,13].

With the potential to improve both the energy density and power density that traditional supercapacitors now struggle with, asymmetric supercapacitors (ASC) have been developed in conjunction with electrical double-layer capacitors (EDLC) and pseudocapacitors [13,14,15]. ASCs are made of a mix of capacitive and battery-type materials. A wide operating voltage window can be supplied to increase energy density without compromising cycle stability by combining the charge storage mechanism of electrostatic adsorption/desorption of EDLC materials with the redox processes of pseudocapacitor materials. To achieve excellent performance of ASCs, carbon materials require large SSA and porous structures for the access of electrolyte and the exchange of ions and electrons. However, the development of metal oxide research has been the main focus of this kind of ASC so far, with activated carbon (AC) being used [8,16,17,18]. This is a result of a number of issues encountered, including poor cycle performance and challenges locating materials that are well-suited as anode and cathode [19,20]. Metal-Organic Frameworks (MOFs) can be made with a variety of controllable structures and have a large specific surface area (SSA), significant pore volume, and adjustable porosity. Thus, they are considered potential materials for supercapacitors [21,22]. However, pristine MOFs typically have poor structural flexibility, low electrical conductivity, and steric hindrance for ion insertion, which leads to lower average electrochemical performance for MOF-based energy storage devices [8]. MOF-derived porous carbons (MDC) and MOF-derived metal oxides (MDMO), on the other hand, have shown exceptional electrochemical performance. For example, Jiang et al. prepared rod-like porous carbon derived from Al-based MOF, and its electrochemical property provides excellent specific capacitance (332 Fg^−1^ at 1 Ag^−1^) [23]. Metal oxides derived from ZIF-67 have been in numerous studies [24,25,26,27,28,29] and MnCo_2_O_4_ derived from bimetallic ZIF-67 showed a very high specific capacitance 1763 Fg^−1^ at 1 Ag^−1^ [26]. Between one-metallic MOFs and mixed-metallic or bimetallic MOFs, the electrochemical performance of MOFs with bimetallic presented more rich redox reactions due to the improved charge transfer between two different metal ions, leading to the high specific capacitance [30]. The anode/cathode electrode materials derived from the same MOF naturally match well because their nanostructures are the same. Wang et al. reported that some ASCs have problems with poor-matched anode/cathode electrode material. The positive and negative electrode materials derived from a single pillared MOF [31] showed a high energy density. MDC/ZnCo_2_O_4_ ASC provided excellent cycle stability and the highest energy density (28.6 Wh/kg) at 100 W/kg of power density [8].

MIL-100 (Fe) has relatively large SSA and high porosity, so it can be considered as a potentially promising material for supercapacitor material. Compared to other metals (such as Co and Cr), the metal center of MIL-100 (Fe), iron, is more affordable and non-toxic, making it a more environmentally friendly material [32,33,34,35,36]. It is also a good candidate for possible industrial production due to its viability for synthesis under ambient pressure [37]. MIL-100 (Fe) was used as a precursor for the supercapacitor electrode material in the previous study and its electrochemical performance resulted in 214 Fg^−1^ at current density 50 mAg^−1^ [32]. However, the fabrication of ASC with MIL-100 (Fe) derivatives has not been reported previously and there is a possibility to improve low capacitance by ASC with MIL-100 (Fe) derivatives.

In this study, MIL100 (Fe), manufactured using three distinct Fe sources, was converted into MOF-derived carbons (MDCs) with single narrow pores or micro- and mesopore structures through carbonization and acid treatment. Additionally, an annealing procedure converted a precursor surface into MDMO (MOF-derived metal oxide) α-Fe_2_O_3_. The obtained porous carbon (MDC-A nitrate) results in a large SSA (780.4 m^2^g^−1^) and has high specific capacitance of 210.1 Fg^−1^ at 0.1 Ag^−1^. MDMO iron for pseudocapacitor electrode material exhibits superior specific capacitance of 383.1 Fg^−1^ at 0.1 Ag^−1^. These two derivatives were used as working electrode materials to fabricate ASC with KOH/PVP gel electrolyte. ASC resulted in improved capacitance, energy density, power density, and longer lifecycle than SSC. ASC with MDC-A nitrate and MDMO iron derived from MIL-100 (Fe) overcame the limits in the low capacitance. The ASC system will be a promising device for high-performance supercapacitors.

## 2. Materials and Methods

### 2.1. Materials

The chemicals in this study were commercially available and used without further purification. Trimesic acid (BTC), metallic iron powder, iron (III) nitrate nonahydrate (Fe(NO_3_)_3_⋅9H_2_O), Iron (II) chloride tetrahydrate FeCl_2_·4H_2_O, Sodium hydroxide pellets (NaOH), and 69% hydrofluoric acid (HF) were purchased from Sigma-Aldrich (St. Louis, MI, USA). 1M hydrochloric acid (1M HCl), and 68.0–70.0% nitric acid (HNO_3_) were obtained from Samchun Chemicals (Seoul, Republic of Korea). Nickel (Ni) foam (porosity 93%) with 0.9 mm thickness was purchased from Goodfellow (Huntingdon, UK).

### 2.2. Synthesis of MIL-100 (Fe) Metal-Organic Frameworks

The experimental synthesis process is based and revised on previously described sustainable techniques [38,39,40].

#### 2.2.1. MIL-100 (Fe) Iron

In the mixture of 0.85 mL of HF, 0.65 mL of HNO_3_, and 120 mL of DI water, 1.35 g of iron metal powder and 3.4 g of trimesic acid was dissolved, and the mixture was then sonicated for one hour. The mixture was then put into an autoclave lined with Teflon, heated for 24 h at 150 °C, and then cooled to room temperature. The mother liquor was centrifuged at 8000 rpm for 30 min to separate the brown suspension. For purification, 800 mL of slight boiling water at 90 °C was used, together with 5 g of the separated brown suspension, to boil for 5 h while being continuously stirred at 400 rpm. The mixture was then recovered by centrifugation at 8000 rpm. With DI water, it was washed three times. The brown suspension was then poured into 800 mL of EtOH and stirred continuously at 400 rpm for 10 h at 80 °C. By using centrifugation, the brown suspension was separated, and it was then dried at 150 °C overnight. After some time, a brown powder was gathered and identified as MIL-100 (Fe) iron.

#### 2.2.2. MIL-100 (Fe) Nitrate

Fe(NO_3_)_3_⋅9H_2_O (4.04 g) and trimesic acid (1.35 g) were dissolved in 10 mL of DI water. The mixture was first placed in a Teflon-lined autoclave for hydrothermal reaction under autogenous pressure for 12 h at 150 °C, after which it was swirled continuously for 1 h at 400 rpm. Centrifugation at 8000 rpm for 30 min separated the brown suspension from the mother liquor in a Teflon-lined autoclave. It was labeled as MIL-100 (Fe) nitrate after going through the same purification procedures as when making MIL-100 (Fe) iron.

#### 2.2.3. MIL-100 (Fe) Chloride

Trimesic acid (3.352 g) was dissolved in 50 mL of 1M NaOH aqueous solution (1st solution). Then 4.52 g of FeCl_2_·4H_2_O was dissolved in 200 mL of DI water (2nd solution). The first solution was added dropwise over the second solution while it was still being agitated, and the two solutions were constantly stirred at 400 rpm for 30 min. The mixture was agitated at 60 °C for 24 h before being centrifuged at 8000 rpm for 30 min to recover the material. MIL-100 (Fe) chloride was labeled after being purified using the same procedures as during the manufacture of MIL-100 (Fe) iron.

### 2.3. Preparation of MOF-Derived Carbons (MDC) and MOF-Derived Metal Oxide Materials (MDMO)

The synthesized MIL-100 (Fe) iron, nitrate, and chloride were used to prepare two kinds of MDC and MDMO. MDC-A was fabricated for micro- and broad mesopore carbon. MDC-B was produced for narrow micropore carbon.

The precursor MOF was calcined at 800 °C at a heating rate of 5 °C min^−1^ for 3 h in an atmosphere of N_2_, then allowed to cool naturally to ambient temperature. This sintered material was washed by 300 mL of 3 M hydrogen chloride at boiling temperature for one hour while being stirred to remove the iron oxide in order to produce MDC-A. After that, the precipitate was filtered and rinsed with 600 mL of deionized water. A vacuum oven was used to heat the porous carbon overnight at 90 °C. It was designated as sample MDC-A.

The calcination stage was carried out the same way for MDC-B as it was for MDC-A. The iron oxide was then removed from the sintered material by dispersing it in 300 mL of 1 M hydrogen chloride at boiling temperature for 16 h while stirring. After that, the precipitate was filtered and rinsed with 600 mL of deionized water. A vacuum oven was used to heat the porous carbon overnight at 90 °C. It was designated as sample MDC-B.

The precursor MOF was calcinated for 6 h at 350 °C with a heating rate of 5 °C min^−1^, then allowed to cool naturally to ambient temperature. It was designated as an MDMO sample.

### 2.4. Materials Characterization

The morphologies of MIL-100 (Fe) iron, nitrate, and chloride and MDC-A, MDC-B, and MDMO were investigated by scanning electron microscopy (SEM, JSM-6701F, JEOL, Tokyo, Japan). The chemical elements were examined by X-ray photoelectron spectroscopy (XPS, K-Alpha, Thermo Fisher, Waltham, MA, USA) using Al-K*α* radiation. The crystal structures were characterized by X-ray diffraction (XRD) analysis (XRD-6100, Shimadzu, Kyoto, Japan) with Cu-K*α* radiation in the 2θ range of 5–80° with a 5° min^−1^ scan rate. Raman spectra were measured on a Renishaw inVia Reflex Raman Microscope operated at 532 nm. Thermo gravimetric analyzer (TGA-Q500, TA Instruments, New Castle, DE, USA) was used to investigate the thermal stability. Approximately 20 mg of the sample was heated at a heating rate of 5 °C min^−1^ between 20 and 900 °C under N_2_ flow. Pore properties and the specific surface area were investigated by the multipoint Brunauer-Emmett-Teller (BET) method at 77 K using MicrotracBEL (BELSORP MAX). Prior to measurement, samples were degassed at 150 °C for 6 h in a vacuum condition.

### 2.5. Electrochemical Measurements

The electrochemical workstation (CHI760e, CH Instruments, Austin, TX, USA) with a three-electrode system was used to test and evaluate the electrochemical capacitive performance of the MOF-derived electrode materials in a 6 M KOH aqueous electrolyte solution under ambient circumstances. Then, 80 wt% of the working materials, 10 wt% acetylene black, and 10 wt% PTFE were mixed and ground for 30 min to fabricate the working electrode, which was then tableted to the 0.9 mm thick Ni foam. A pressing machine applied 1 MPa of pressure for 10 s to an electrode film (1 × 1 cm^2^) that was placed on the Ni foam of the same size. A three-electrode system comprised a working electrode (1 × 1 cm^2^), Hg/HgO reference electrode, and a 37 mm filament type Pt counter electrode, and 6.0 M KOH electrolyte was used for the electrochemical testing.

For the experiments of the two-electrode system, working electrodes were fabricated into the asymmetric (ASC) and symmetric supercapacitors (SSC) with the alkaline-PVA gel electrolyte. Then, 4.0 g KOH and 4.0 g PVA powder were mixed with 50 mL deionized water under stirring at 300 rpm for 2 h. Then the mixed solution was heated to 100 °C and turned to the transparent solution. KOH-PVA gel-like solution was dipped into the one side of each working electrode and dried at room temperature until the solvent evaporated. After that, two working electrodes were stacked on the gel electrolyte loaded side on another loaded side.

Cyclic voltammetry (CV) and galvanostatic charge-discharge (GCD) techniques were used to assess the electrochemical characteristics in the three-electrode system in the voltage window of 1 to 0 V for MDC-A and MDC-B and −1 to −0.5 V for MDMO. In a three-electrode system, the gravimetric specific capacitance ($C_{\mathrm{three}}$, F/g) of a single electrode was measured in 6 M KOH. Two-electrode systems from the CV (voltage window 0.8 to 1.6 V at 100 mVs^−1^), GCD (voltage window range: 0–1.2 V for ASC and 0–0.8 V for SSC at the current density: 0.1–5 Ag^−1^), and EIS (0.01–100,000 Hz) were studied.

The specific capacitance in the three-electrode system was calculated from GCD by the following equation:

(1) $$C_{\mathrm{three}}=\left(I∆t\right)/\left(m∆V\right)$$

where C_three_ (F g^−1^) is the specific capacitance of MDC-A, MDC-B, and MDMO tested in the three-electrode system; I (A) is the discharge current; Δt (s) is the discharge time; ΔV (V) is the potential window; m (g) is the mass of MDC-A, MDC-B, and MDMO.

The specific capacitance in the two-electrode system was calculated from GCD by the following equation:

(2) $$C_{\mathrm{two}}=\left(I∆t\right)/\left(m∆t\right)$$

(3) $$E=0.5{\;C}_{\mathrm{two}}∆V^{2}/3.6$$

(4) P=3600 Energy density/∆t

where $C_{\mathrm{two}}$ (F g^−1^) is the specific capacitance of working material-based solid supercapacitor; I (A) is the discharge current; Δt (s) is the discharge time; ΔV (V) is the voltage window; m (g) is the total mass loading of the active materials on the two electrodes; E (Wh kg^−1^) is the average energy density; and P (W kg^−1^) is the average power density.

## 3. Result and Discussion

### 3.1. Material Morphology and Structural Characterization

**Figure 1:**
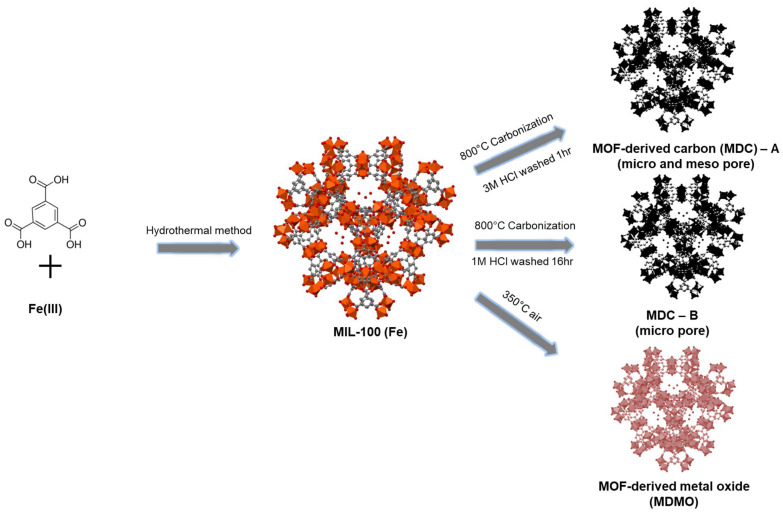
The schematic illustration of MDC-A, MDC-B, and MDMO from MIL-100 (Fe).

The hydrothermal approach is used to synthesize three MIL-100 (Fe) precursors by reacting three different kinds of iron metal salts with H_3_BTC. Figure 1 shows the schematic preparation procedure for MDC-A, MDC-B, and MDMO using MIL-100 (Fe) iron, nitrate, and chloride. Two primary techniques are used in the synthesis process. First, during the carbonization process, releasing CO_2_ from the bond between –COO- in ligand and Fe(III) metal can create uniform pores in the resulting carbon materials. Furthermore, carbonized MIL-100 (Fe) maintains the distinctive structure derived from the mother MIL-100 (Fe) during the carbonization process. The α-Fe_2_O_3_ particles (intermediate product) can be removed after a slight boiling in a hydrochloric acid solution with varying concentrations and periods, increasing the number of pores with a specific dimension and ultimately producing MDCs with reduced diameters. Mesoporous α-Fe_2_O_3_, MOF-derived metal oxide (MDMO), is created after the calcination of MIL-100 (Fe) in air atmospheric conditions.

**Figure 2:**
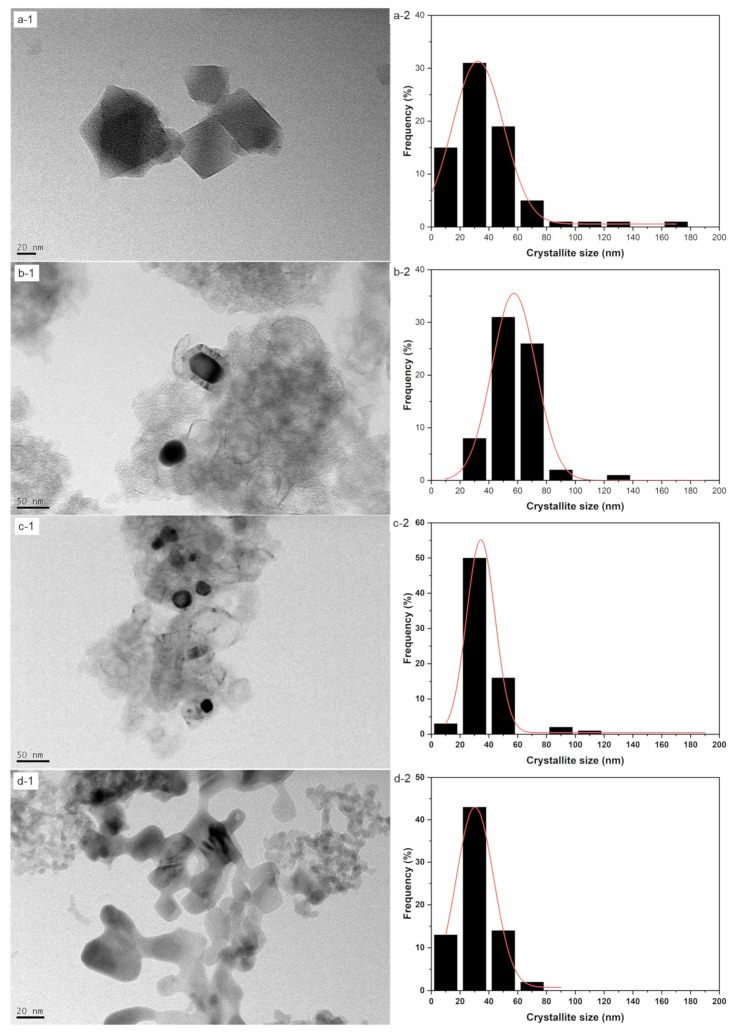
TEM images (-1) and crystallite size distributions (-2): (**a**) MIL-100 (Fe) nitrate; (**b**) MDC-A nitrate; (**c**) MDC-B nitrate; (**d**) MDMO nitrate.

MIL-100 (Fe), MDC-A, MDC-B, MDMO morphologies, and elemental compositions were investigated by SEM and XPS. As shown in Figure 2, Figures S4 and S6, all MIL-100 (Fe) showed an irregular cubic-like structure with a relatively clean and smooth surface. The size of the synthetic MIL-100 (Fe) with an iron powder was approximately 459.7 nm in the average crystallite size, while the average crystallite size of the MOF synthesized with nitrate and chloride precursors was approximately 32.4 nm and 212.3 nm, respectively. These three MIL-100 (Fe) precursors showed similar results and forms to those in the literature [38,39,40]. MDC-A and MDC-B were obtained by washing in different concentrations and times after carbonization at the same temperature, showing similar morphologies to the parent MOFs (Figure 2 and Figure S4). However, there were some distortions in their geometry with a rough surface. Moreover, some noticeable pores can be observed. In contrast, the MDMO generated at 450 °C under air clearly shows that the surface is rough, as seen in Figure 2d and Figure S4, and that the α-Fe_2_O_3_ nanoparticles have been generated.

**Figure 3:**
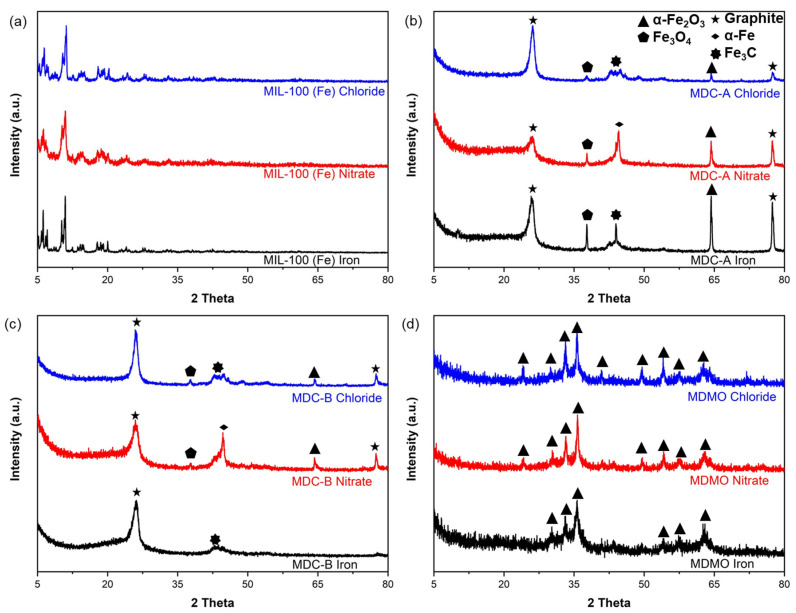
XRD patterns of MIL-100 (Fe) synthesized with different iron sources (**a**); MDC-A (**b**); MDC-B (**c**); MDMO (**d**).

Figure 3a–d displays the XRD patterns for all MIL-100 (Fe), MDC-A, MDC-B, and MDMO. The X-ray powder diffraction patterns of three different types of MIL-100 (Fe) synthesized in this study were well-concordant with MIL-100 (Fe) in previous studies [38,39,40]. After the carbonization and acid washing processes (Figure 3b,c), the distinctive diffraction peaks of MIL100 (Fe) disappear, showing new diffraction lines that are attributed to graphitic carbon (2θ = 26.5° and 77.4°) [41]. Another identifiable signal for MDC-A and MDC-B nitrate can be seen at 2θ = 44.7°, which denotes the presence of α-Fe (JCPDS, No. 87-0722) [42]. Contrarily, only the samples of MDC-A and MDC-B iron and chloride show the existence of Fe_3_C (2θ = 42.9°, 43.7°, and 45.7°). The Fe incorporated in the MIL-100 (Fe) structure could be heated to form isolated Fe species throughout the synthesis, and at a higher temperature, a portion of these species reacted with carbon to produce Fe_3_C particles. The reduction of some organic byproducts may be the key to the absence of Fe_3_C [43]. Its electrocatalytic activity may be enhanced by producing Fe/Fe_3_C species by accelerating the graphitization of carbons at high temperatures [44,45]. The peak intensity at 37.6° is attributed to Fe_3_O_4_ (JCPDS No. 01-089-2335). After high-temperature pyrolysis, the carbon matrix would be treated with acid to create porosity by eliminating some of the Fe/Fe_3_C species within. XRD peaks of MDMO in Figure 3 agreed well with the previous study [46], indicating that synthesized MDMO is pure α-Fe_2_O_3_ (JCPDS No. 33-0664). None of the samples show any additional peaks, proving that the three MDMOs are well-crystallized α-Fe_2_O_3_.

**Figure 4:**
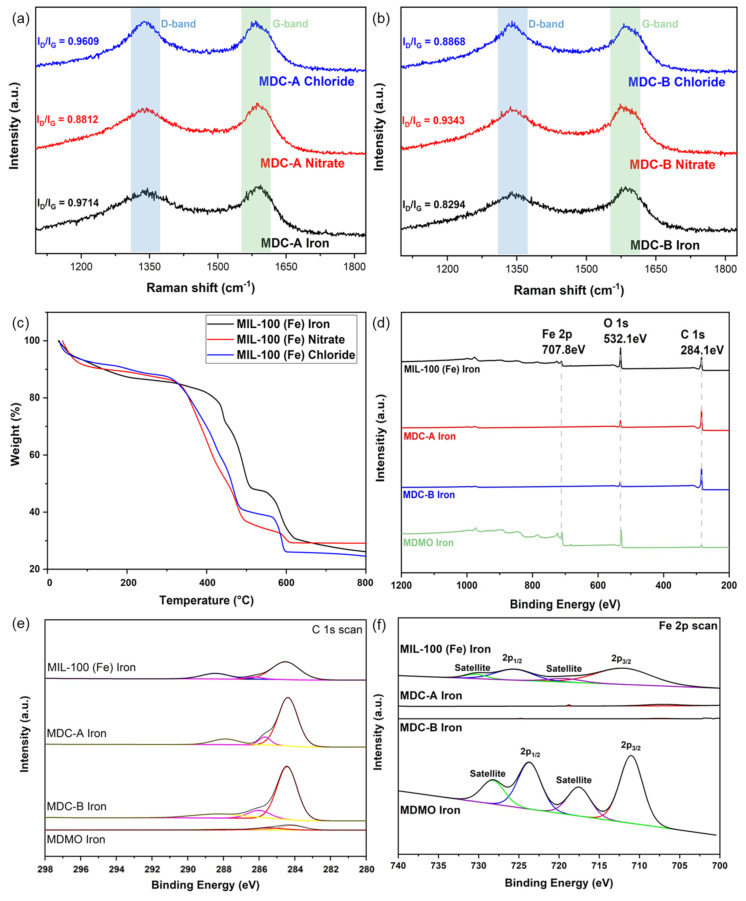
Raman spectra of MDC-A (**a**), MDC-B (**b**); TGA data of MIL-100 (Fe) iron, nitrate, chloride (**c**); Wide-scan XPS (**d**), C 1s (**e**) and Fe 2p spectra (**f**) of MIL-100(Fe) iron, MDC-A, MDC-B, and MDMO.

Raman spectra were used to examine the defect density of two different types of MDC. The D band (1358 cm^−1^) and G band (1580 cm^−1^) distinctive peaks may be recognized clearly in Figure 4a,b. The high I_D_/I_G_ value suggests that the hydrochloric acid boiling procedure can produce structural flaws and more mesopores in the carbon material made from MIL-100 (Fe), increasing surface area and ultimately improving electrochemical performance [47].

Three types of MIL-100 (Fe) were subjected to a thermogravimetric analysis in a nitrogen atmosphere (Figure 4c). The weight loss in the first segment (25–300 °C) is caused by water that has been adsorbed and other solvents (like ethanol) employed during the synthesis method. The overall mass weight losses for MIL-100 (Fe) iron, nitrate, and chloride are around 14.39%, 13.43%, and 12.57%, respectively. Since MIL-100 (Fe) iron is more microporous than the other two samples, more bound water may be present in the frameworks, which causes the first segment to lose weight the fastest [2,47]. This result is consistent with the pore structural properties results (Table 2). The partial collapse of MIL-100 (Fe) frameworks causes the second significant loss in the temperature range of 300–550 °C, which accounts for roughly 52 wt% for all three samples. The final loss in the 550–750 °C region results from completely degrading organic linkers in MIL-100 (Fe) [2,47]. 

The chemical compositions of all MIL-100 (Fe), MDC-A, MDC-B, and MDMO were investigated by XPS analysis. According to the preliminary experiments, the surface elemental compositions of MIL-100 (Fe) and its derivatives have the same results, so only the MIL-100 (Fe) iron and MDC-A, MDC-B, and MDMO derived from MIL-100 (Fe) precursors are presented here. The wide-scan XPS spectra of C 1s and Fe 2P exhibit obvious variations, as illustrated in Figure 4d–f. Three peaks with corresponding centers at 284.5, 288.4, and 286.3 eV are seen in the high-resolution C 1s spectra of MIL-100 (Fe). The bonds of the phenyl and carboxyl signals are represented by the peaks at 284.5 and 288.4 eV, respectively [48,49]. The carbon on the MIL-100 (Fe) surface is responsible for the following peak, which is positioned at 286.3 eV [50]. The peak at 288.4 eV vanishes after carbonization and hydrochloric acid washing, indicating that the carboxyl group has been pyrolyzed and converted into carbon [51]. The Fe 2p spectrum (Figure 4f) can be deconvoluted into four peaks with centers at 711.9, 717.7, 725.9, and 731.6 eV, respectively. These peaks correspond to Fe 2p_1/2_ and Fe 2p_3/2_, as well as the two satellite peaks of Fe 2p_3/2_ and Fe 2p_1/2_, which are similar to those previously reported for α-Fe_2_O_3_ derived from MIL-100 (Fe) [45]. Table 1 shows the precise elemental makeup of MIL-100 (Fe) iron and its derivatives as determined by the XPS technique. In MDC-A and MDC-B, the surface atomic percent of the element Fe is 0.13% and 0.04%, respectively. The absence of the Fe 2p peak and the close to zero surface atomic percent of the element Fe indicate that the hydrochloric acid boiling process has entirely eliminated all of the Fe atoms from MDC-A and MDC-B. Compared to MDMO iron, the Fe 2P peak has increased significantly to 23.55%, which shows that the calcination process has caused α-Fe_2_O_3_ metal oxides to grow on the surface of the MIL-100 (Fe) precursor. The O 1s peak represents the Fe-O-C species at 530.5 eV in Figure S1 [52]. The relatively higher carbon content of MDMO iron compared to its carbon content precursor MOF is due to the fact that most of the organic ligands that maintain the MOF structure were removed by gasification to CO_2_ and CO during the air heat treatment. Since Fe is not removed during the air atmosphere heat treatment process, the C/Fe ratio of MOF is 6.75 and that of MDMO is 1.84, indicating that a significant amount of carbon has been removed.

**Table 1:** The elemental composition of MIL-100 (Fe) iron and its derivatives tested by XPS.

Samples	C (at.%)	O (at.%)	Fe (at.%)
MIL-100 (Fe) Iron	29.67	55.61	4.39
MDC-A Iron	79.68	10.58	0.13
MDC-B Iron	81.61	8.64	0.04
MDMO Iron	43.35	21.06	23.55

**Figure 5:**
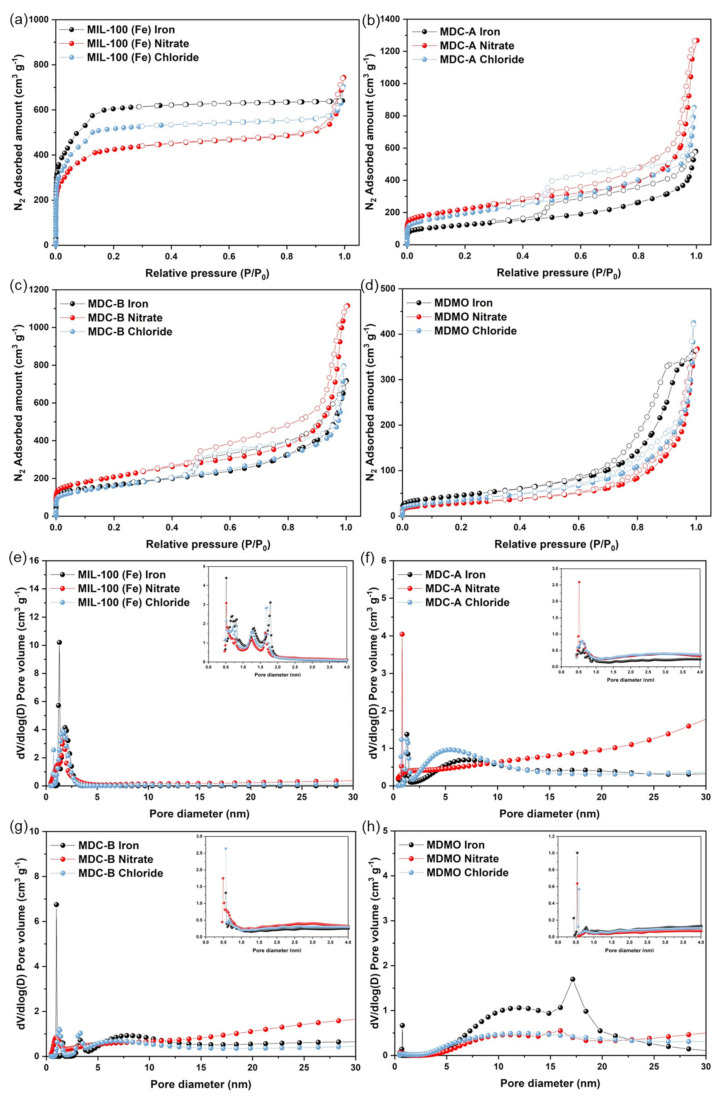
Nitrogen adsorption-desorption isotherm of MIL-100 (Fe) (**a**), MDC-A (**b**), MDC-B (**c**), MDMO (**d**); pore size distribution of MIL-100 (Fe) (**e**), MDC-A (**f**), MDC-B (**g**), MDMO (**h**).

Research on the specific surface area (SSA) and pore size distribution of MIL-100 (Fe), MDC-A, MDC-B, and MDMO is done using N_2_ adsorption-desorption studies at 77K. The nitrogen adsorption isotherms of all MIL-100 (Fe) samples follow the typical II type curve shown in Figure 5a. The sharp rise at high pressure (P/P_0_ = 1) and the steep rise at a low relative pressure (P/P_0_ < 0.001) indicate the presence of micro- and macroporous structures, respectively. Additionally, the hysteresis loops indicated the mesoporous structure with aggregates of plate-like particles giving rise to slit-shaped pores for MDC-A, MDC-B, and MDMO by the hysteresis loops (P/P_0_ = 0.4–1.0) that can be classified as H3 type. The BET SSA and pore volume of derivatives have decreased compared to MIL-100 (Fe) progenitors and their derivatives. However, MDC-A and MDC-B nitrate pores have slightly increased in size (Table 2). In Figure 5f–h, the pore size distribution curve of derivatives exhibits micro-mesoporous distribution with sharp peaks centered at 1 nm, and broad peaks centered at about 5–15 nm. By performing carbonization and HCl washing processes, the average pore size of derivatives demonstrated an increase in pore diameter.

**Table 2:** Structural properties of MIL-100 (Fe) iron, nitrate, chloride, and its derivatives calculated from N_2_ adsorption at 77 K.

Samples	S_BET_ (m^2^g^−1^)	V_total_ (cm^3^g^−1^)	V_micro_ (cm^3^g^−1^)	V_meso_ (cm^3^g^−1^)	V_meso_/V_total_ (%)
MIL-100 (Fe) iron	2147.1	0.9865	0.8180	0.1685	17.08
MIL-100 (Fe) nitrate	1493.3	0.8342	0.5936	0.2406	28.84
MIL-100 (Fe) chloride	1824.3	0.8885	0.7081	0.1804	20.30
MDC-A iron	432.2	0.5639	0.1667	0.3972	70.44
MDC-A nitrate	780.4	0.9840	0.2998	0.6842	69.53
MDC-A Chloride	686.9	0.7848	0.2526	0.5322	67.81
MDC-B iron	575.2	0.7367	0.2274	0.5093	69.13
MDC-B nitrate	735.7	0.9650	0.2789	0.6861	71.10
MDC-B Chloride	567.9	0.6742	0.2110	0.4632	68.70
MDMO iron	162.6	0.5153	0.0597	0.4556	88.42
MDMO nitrate	101.7	0.2857	0.0369	0.2488	87.08
MDMO chloride	127.4	0.3269	0.0442	0.2827	86.50

Brunauer-Emmett-Teller (BET) and NLDFT were used to compute the SSA and porosity characteristics of MIL-100 (Fe) iron, nitrate, chloride, and their derivatives. The results are shown in Table 2. These show that MIL-100 (Fe) pores are formed and that a single MOF can yield MDC and MDMO. It is anticipated that the MDC and MDMO will exhibit outstanding electrochemical performance in supercapacitors since the big SSA and high porosity are always connected with the outstanding electrochemical performance of electrode materials.

### 3.2. Electrochemical Performance

**Figure 6:**
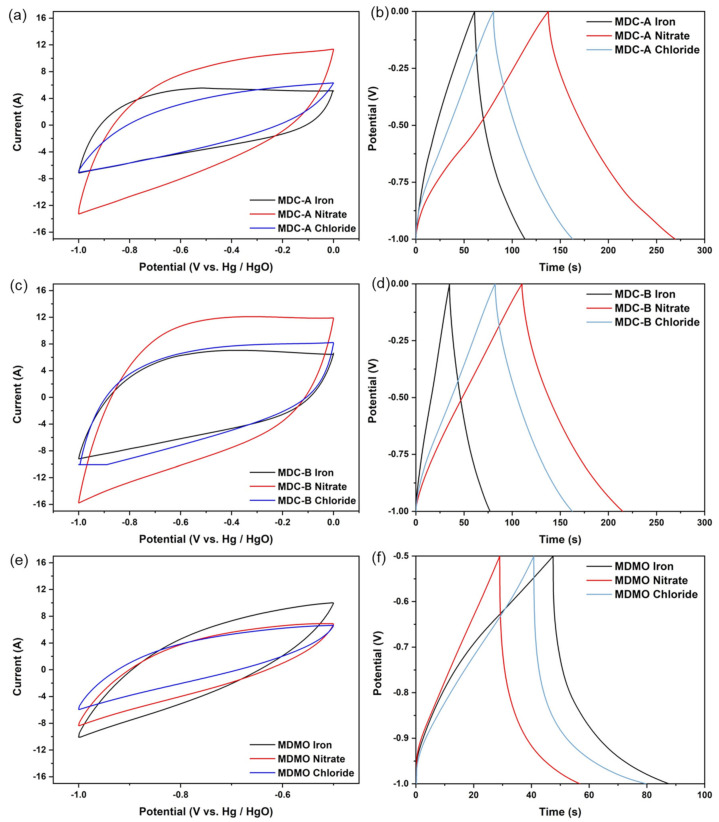
The three-electrode electrochemical performances of MDC and MDMO: CV curves comparison at 200 mVs^−1^ of (**a**) MDC-A, (**c**) MDC-B, (**e**) MDMO; GCD curves at 1 Ag^−1^ of (**b**) MDC-A, (**d**) MDC-B, (**f**) MDMO.

The as-obtained MIL-100 (Fe)-derived carbon (MDC) and metal oxide (MDMO) were investigated as potential supercapacitor electrode material. The three-electrode system used an aqueous 6 M KOH as the electrolyte and conducted cyclic voltammetry (CV) and galvanostatic charge-discharge (GCD) tests to methodically study the electrochemical performances of two types of MDCs and MDMO electrodes. The potential ranges of −1.0 to 0.0 V and −1.0 to −0.5 V (vs. Hg/HgO) were used to test the CV investigations for the electrodes based on MDC and MDMO, respectively. The capacitive characteristics of two MDCs and an MDMO at the scan rate 200 mVs^−1^ are shown in Figure 6a,c,e (Figure S2 shows various scan rates from 5 to 200 mVs^−1^). The CV curves of MDC-A, MDC-B, and MDMO exhibit symmetry shapes. They can be maintained at a high scan rate 200 mVs^−1^ without noticeably changing or distorting, demonstrating their exceptional rate capacity. This excellent rate performance is attributed to its many mesopores, which are produced from the unique MOF structure and enable the transport of electrolyte ions in the pore channels [8]. In the case of MDMO-based electrodes, Figure 6e makes it abundantly clear that none of the three different MDMOs exhibit a pair of glaring redox peaks, as reported by earlier studies [53,54]. Similar phenomena happen when the materials are calcined, and α-Fe_2_O_3_ is formed on their surface.

In Figure S3, the GCD curves of the MDC-A, MDC-B, and MDMO-based electrodes are shown for a range of current densities from 0.3 to 10 Ag^−1^. Regarding MDC-A and B, the GCD curves always maintain symmetric triangular shapes at all current densities and do not show an obvious IF drop at the initial discharge stage, indicating the presence of an electrical double-layer capacitor made of carbon materials. According to the GCD and CV data, the MDC-A and MDC-B have exceptional columbic efficiency and good capacitive behavior when utilized as supercapacitor electrodes. The probable electrochemical redox reactions in KOH for MDMO-based electrodes can be represented by Equation (5) [55]:

(5) $${\mathrm{Fe}}_{2}O_{3}\;+\;2K^{+}\;+\;2e^{-}\;↔\;K_{2}{\mathrm{Fe}}_{2}O_{3}$$

**Figure 7:**
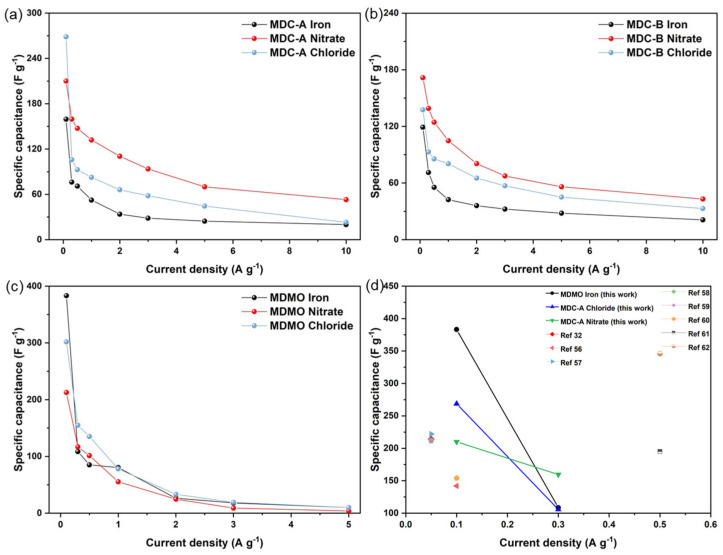
Specific capacitances at different current densities (0.1–10 Ag^−1^: (**a**) MDC-A, (**b**) MDC-B, (**c**) MDMO; comparison of specific capacity with other literature (**d**).

Figure 7a–c shows the mass-specific capacitance derived from GCD curves using Equation (1) at various current densities. This figure shows the individual capacitances for MDC-A, MDC-B, and MDMO. For porous carbon electrode, MDC-A derived from the MIL-100 (Fe) nitrate precursor has the most significant specific capacitances 210.1, 159.6, and 139.1 Fg^−1^ at current densities 0.1, 0.3, and 10 Ag^−1^, respectively. The highest specific capacitance for metal oxide electrode is displayed by MDMO iron, which is 383.1 Fg^−1^ at 0.1 Ag^−1^ and Fg^−1^ at 0.1 Ag^−1^. At low current densities, MDMC has a higher capacitance than MDC-A and MDC-B. These capacitances at each corresponding current density are almost higher than those of the derived materials using MOF as precursors in the literature (Figure 7d) [32,56,57,58,59,60,61,62].

MDC-A nitrate is selected as the negative electrode material for ASC. Between MDC-based electrodes, this material has a large SSA (780.4 m^2^g^−1^) with micro- and mesopore, and exhibits excellent specific capacitance at current densities 0.3 to 10 Ag^−1^. EDLCs employ the principle of forming electric double layers to store energy, in which cations and anions accumulate between the electrode and the electrolyte, creating a thin electric double layer. Energy is stored and supplied through the charging and discharging processes of these electric double layers. Utilizing electrode materials with a large specific surface area enables the formation of electric double layers on a larger surface area, thus increasing the amount of charge that can be stored [23,32,51]. The pseudocapacitors store energy by quickly exchanging electrons with electrode materials on their surface. The surface of the electrode material has a direct impact on how well the shootout capacitor performs. The surface area represents the electrode material’s fine structure, which offers more active areas and greater room for electrochemical processes to take place [3,53,54]. Although MDMO iron-based electrode has lower specific capacitance at the current densities 0.3 to 1 Ag^−1^, it results in equivalent specific capacitance after 1 Ag^−1^ and provides a larger SSA (162.6 m^2^g^−1^) than the other MDMO materials. SSA has a direct impact on specific capacitances. As can be seen in Figure 7, the specific capacitance is higher when the SSA is larger. For materials with similar SSAs, those with a lower proportion of mesopores in pore volume also have higher specific capacitances.

**Figure 8:**
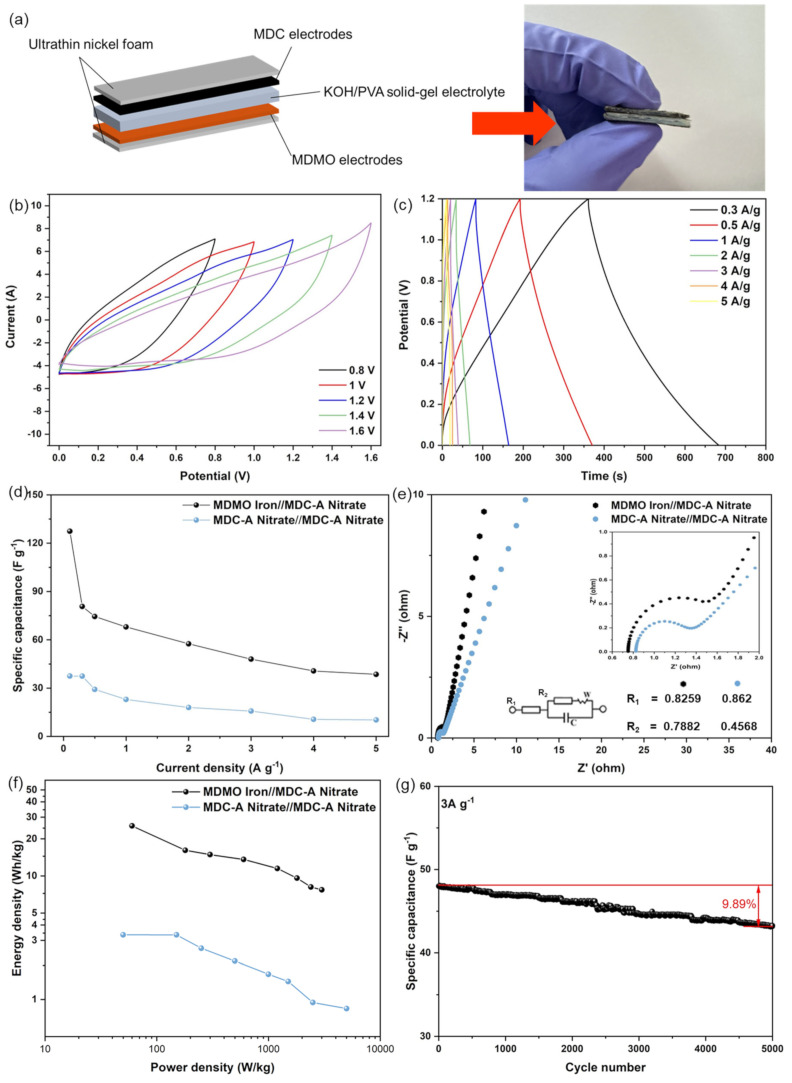
Electrochemical performances of asymmetric and symmetric supercapacitors in a two-electrode device with KOH/PVA electrolyte: (**a**) Optical photograph and illustration of the fabrication of solid—state supercapacitors; (**b**) CV curves collected in different voltage windows; (**c**) GCD curves at different current densities (0.3–5 Ag^−1^); (**d**) Specific capacitance of ASC and SSC at different current densities; (**e**) EIS curves of ASC and SSC; (**f**) Ragone plots of the assembled supercapacitors in this study; (**g**) cycling performance of ASC at 3 Ag^−1^.

In order to further accurately determine the feasibility of the practical applications of MIL-100 (Fe)-derived materials in electrochemical energy storage, KOH/PVA electrolyte was used to examine the capacitive performance of ASC and SSC (Figure 8a). The positive electrode materials were MDMO iron and negative electrode materials were MDC nitrate in ASC. The SSC was also created simultaneously using two MDC-A nitrate electrodes and assessed for comparability. The capacitive capabilities with ASC and SSC were further examined using CV curves, GCD, and EIS measurements. Only the CV and GCD results of the ASC device are presented here because preliminary experiments show that it has significantly better capacitive performance than the SSC. The CV curves at 100 mVs^−1^ are shown in Figure 8b for various scan voltage windows (0.8 V to 1.6 V). The CV curves can maintain the shape when the voltage window is below 1.2 V. The CV curves below 1.4 V and 1.6 V show evident distortion, proving that 1.2 V is the optimal voltage window for this ASC with KOH/PVA gel electrolyte. In comparison, the SSC has a voltage window of 0.0 to 0.8 V. The voltage window in ASC is higher and has a more stable performance than SSC, indicating derivatives from a single MOF have unique advantages [62,63]. Additionally, GCD measurements were made at various current densities ranging from 0.3 to 5 Ag^−1^ and are depicted in Figure 8c for a potential window from 0.0 to 1.2 V. Even at a high current density of 5 Ag^−1^, the initial voltage loss (IR drop) is not shown in the discharge curves, pointing to a quick I-V response and meager internal resistance of the ASC [64], which the EIS data will validate.

Equation (2) was used to calculate the specific capacitance of the SSC (MDC-A nitrate/MDC-A nitrate) and ASC (MDMO iron/MDC-A nitrate). The specific capacitances of these two devices at various scan rates are shown in Figure 8d. It is obvious that the specific capacitance of the ASC is substantially higher than that of the SSC. At current densities of 0.1 Ag^−1^ and 3 Ag^−1^, respectively, the specific capacitance of ASCs can reach maximum values of 127.4 Fg^−1^ and 48.0 Fg^−1^. The EISs of ASC and SSC were also studied and shown with an inset of the equivalent circuit diagram in Figure 8e. Due to their exact compatibility, ASC has the lowest internal resistance. R1 values of 0.8259 Ω and 0.862 Ω for ASC and SSC, respectively, can be found. ASC also has a medium charge-transfer resistance (R2) of 0.7882 Ω. This means that ASC indicates exceptional electrical conductivity, excellent ion diffusion, and rapid charge transfer in the process of charge/discharge.

The Ragone plot (power density vs energy density) is a direct performance standard in energy storage devices to evaluate the performance of the as-assembled ASC and SSC. Figure 8f shows the Ragone plots for the ASC and SSC. Equations (3) and (4) are used to calculate the energy and power densities. It is discovered that the ASC can maintain 7.7 Wh/kg at 3 kW/kg while delivering a maximum energy density of 25.5 Wh/kg at a power density of 60 W/kg. The SSC, on the other hand, has a significantly lower energy density for the same power density. SSC has a minimal energy density of 3.3 Wh/kg. These proved that the ASC outperformed.

The following considerations and practical reasons are the key causes for the superior energy storage performance of MDMO iron and MDC-A nitrate asymmetric supercapacitors. Firstly, the MDC and MDMO electrode materials derived from the MIL-100 (Fe) have a porous structure with a significant SSA and an appropriate pore ratio between the micro- and mesopore. These features provide abundant active sites for ion accumulation and pore channels for rapid ion transfer. Another factor is the nanoscale size, which results in a short charge transport channel during the charging/discharging process and leads to outstanding rate performance. Lastly, the ASC features a broad 1.2 V working voltage window, and its energy density is noticeably increased by its particular capacitance and matchable voltage window. At a current density of 3 Ag^−1^, as depicted in Figure 8g, the cycling performance of the ASC was examined. Additionally, the specific capacity retention rate of the ASC is approximately 90.11% of its initial value after 5000 charge-discharge cycles, indicating excellent cycle performance. According to these experimental data, the ASC fabricated with MDC-A and MDMO derived from the MIL-100 (Fe) has promising potential in usable energy storage.

## 4. Conclusions

In summary, three metal precursors (iron powder, nitrate, and chloride) and trimesic acid were prepared to synthesize MIL-100 (Fe). For supercapacitor applications, micro- and mesoporous carbon MDC-A and microporous carbon MDC-B were synthesized by carbonization and acid washing processes, and α-Fe_2_O_3_ metal oxides were obtained by calcination in air. The large surface area and porosity led to excellent electrochemical performances; MDC-A nitrate and MDMO iron provided specific capacitance 210.1 and 383.1 Fg^−1^ at 1 Ag^−1^, respectively. These two electrode materials are chosen to fabricate ASC and SSC with KOH/PVA gel electrolyte. MDC-A nitrate/MDMO iron-based ASC results in superior battery-like capacitance and rate capacity, which is excellent compared to MDC-A-based SSC. The maximum energy density of ASC shows 25.5 Wh/kg at the power density 60 W/kg, and still maintains 7.7 Wh/kg at 3 kW/kg. The cycling test shows that the rate dropped only 9.89% after 5000 cycles. Considering ASC strategy with MOFs derivatives can improve electrochemical performance and overcome limitations in supercapacitor devices.

## Data Availability

Not applicable.

## Supplementary Materials

The following supporting information can be downloaded at: https://www.mdpi.com/article/10.3390/nano13121824/s1, Figure S1. O 1s spectra of MIL-100(Fe) iron, MDC-A, MDC-B, and MDMO; Figure S2. The three-electrode electrochemical performances of MIL-100 (Fe) derivatives: CV curves at different scan rates (1–200 mVs^−1^): (a) MDC-A iron; (b) MDC-A nitrate (c) MDC-A chloride; (d) MDC-B iron; (e) MDC-B nitrate; (f) MDC-B chloride; (g) MDMO iron; (h) MDMO nitrate; (i) MDMO chloride; Figure S3. The three-electrode electrochemical performances of MIL-100 (Fe) derivatives: GCD curves at different current densities (0.3–5 Ag^−1^): (a) MDC-A iron; (b) MDC-A nitrate (c) MDC-A chloride; (d) MDC-B iron; (e) MDC-B nitrate; (f) MDC-B chloride; (g) MDMO iron; (h) MDMO nitrate; (i) MDMO chloride; Figure S4. SEM images of low (-1) and high magnification (-2): (a) MIL-100 (Fe) iron; (b) MDC-A iron; (c) MDC-B iron; (d) MDMO iron; (e) MIL-100(Fe) nitrate; (f) MDC-A nitrate; (g) MDC-B nitrate; (h) MDMO nitrate; (i) MIL-100 (Fe) chloride; (j) MDC-A chloride; (k) MDC-B chloride; (l) MDMO chloride; Figure S5. SEM images of before cycling test (-1) and after cycling test (-2): (a) MDC-A nitrate; (b) MDMO iron; Figure S6. TEM images (-1) and crystallite size distribution (-2): (a) MIL-100 (Fe) iron; (b) MIL-100 (Fe) chloride.
