# Supplementary material for: Asymmetric Supercapacitors Using Porous Carbons and Iron Oxide Electrodes Derived from a Single Fe Metal-Organic Framework (MIL-100 (Fe))
Source: Nanomaterials (Basel). 2023 Jun 8;13(12):1824. doi: 10.3390/nano13121824 (PMC10301871; doi:10.3390/nano13121824)
Supplement: Supplementary file 1 [file nanomaterials-13-01824-s001.zip › nanomaterials-2401248-supplementary.pdf]

nanomaterials

Supplementary Materials

Asymmetric Supercapacitors Using Porous Carbons and Iron Oxide Electrodes Derived From a Single Fe Metal-Organic Framework

Seong Cheon Kim [1,2], Siyoung Q. Choi[1,3], and Jeasung Park [2,*]

Department of Chemical and Biomolecular Engineering, Korea Advanced Institute of Science and Technology (KAIST), Daejeon, 34141, South Korea
[2] Korea Institute of Industrial Technology (KITECH), 89 Yangdaegiro-gil Ipjang-myeon Seobuk-gu, Cheonan-si, Chungcheongnam-do, 31056, Republic of Korea
[3] KAIST Institute for the Nanocentury, Korea Advanced Institute of Science and Technology (KAIST), Daejeon, 34141 Republic of Korea
* Correspondence: jpark@kitech.re.kr

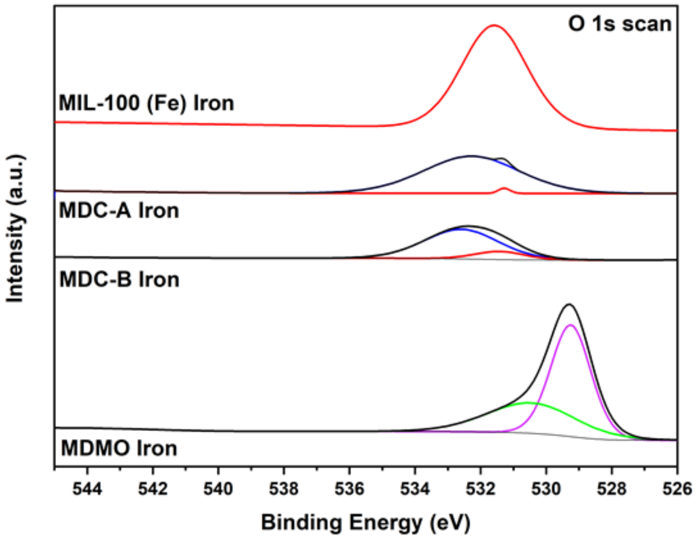

Figure S1. O 1s spectra of MIL-100(Fe) iron, MDC-A, MDC-B and MDMO.

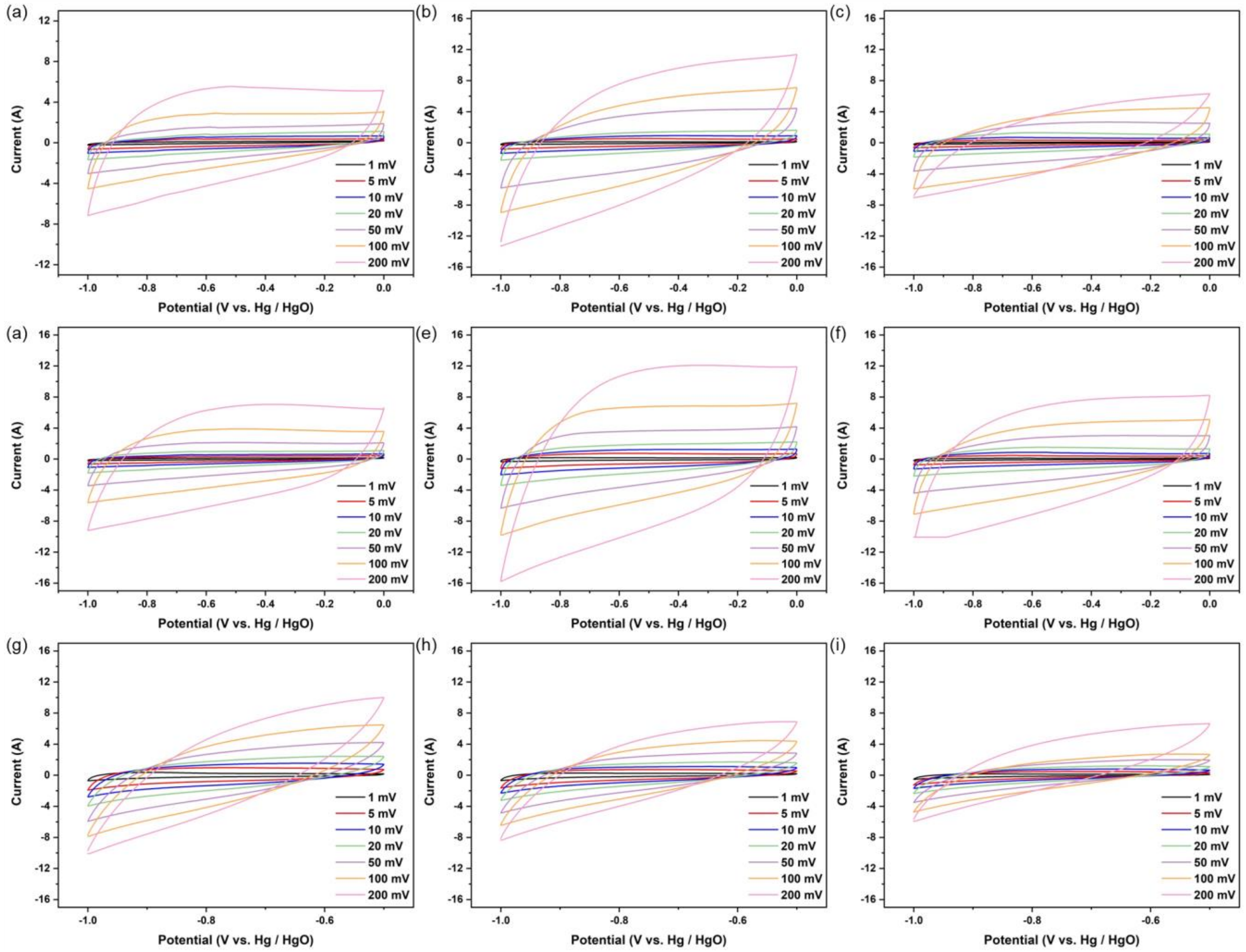

Figure S2. The three-electrode electrochemical performances of MIL-100 (Fe) derivatives: CV curves at different scan rates (1-200 mVs^{-1}) : (a) MDC-A iron; (b) MDC-A nitrate (c) MDC-A chloride; (d) MDC-B iron; (e) MDC-B nitrate; (f) MDC-B chloride; (g) MDMO iron; (h) MDMO nitrate; (i) MDMO chloride.

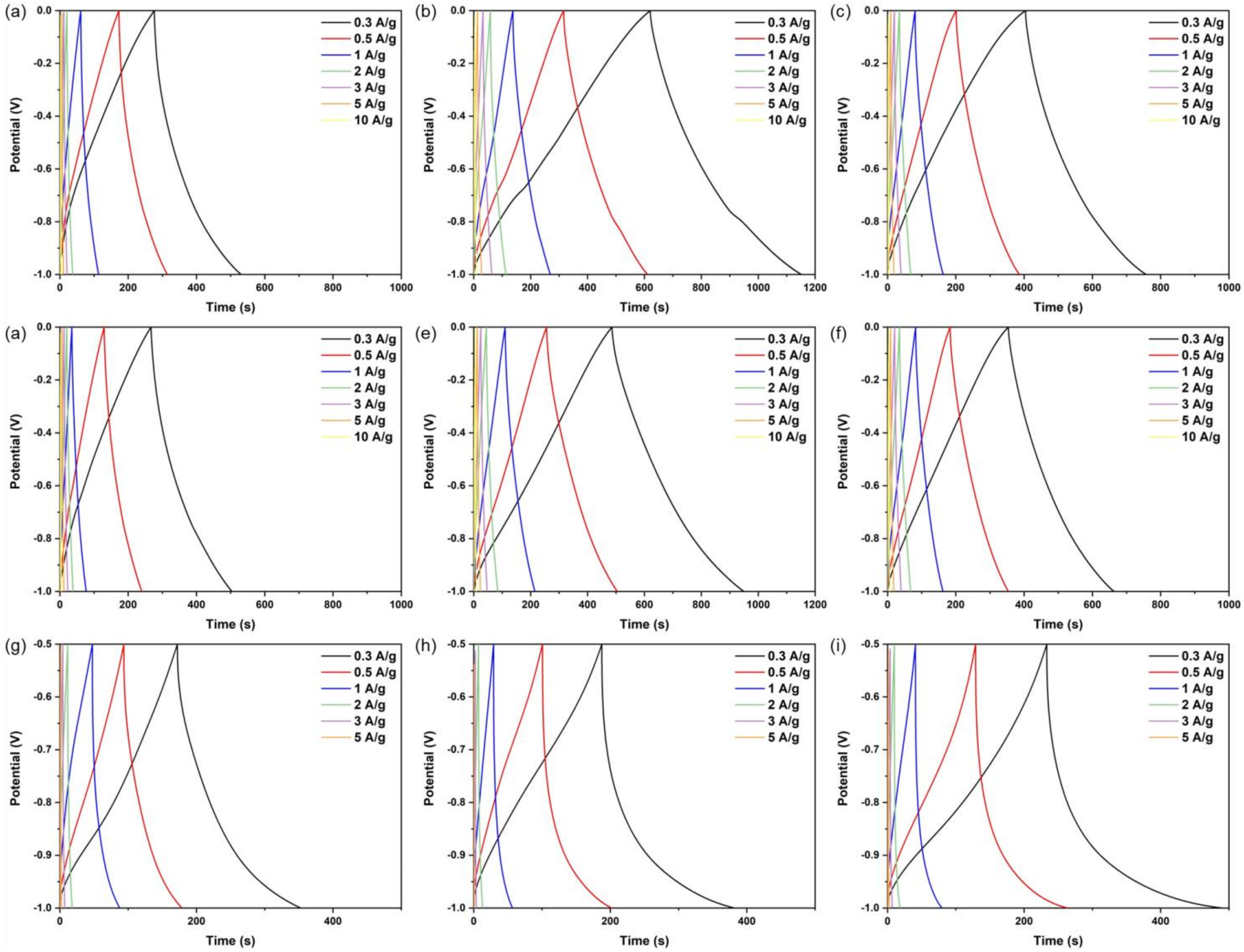

Figure S3. The three-electrode electrochemical performances of MIL-100 (Fe) derivatives: GCD curves at different current densities (0.3-5 Ag^{-1}) : (a) MDC-A iron; (b) MDC-A nitrate (c) MDC-A chloride; (d) MDC-B iron; (e) MDC-B nitrate; (f) MDC-B chloride; (g) MDMO iron; (h) MDMO nitrate; (i) MDMO chloride.

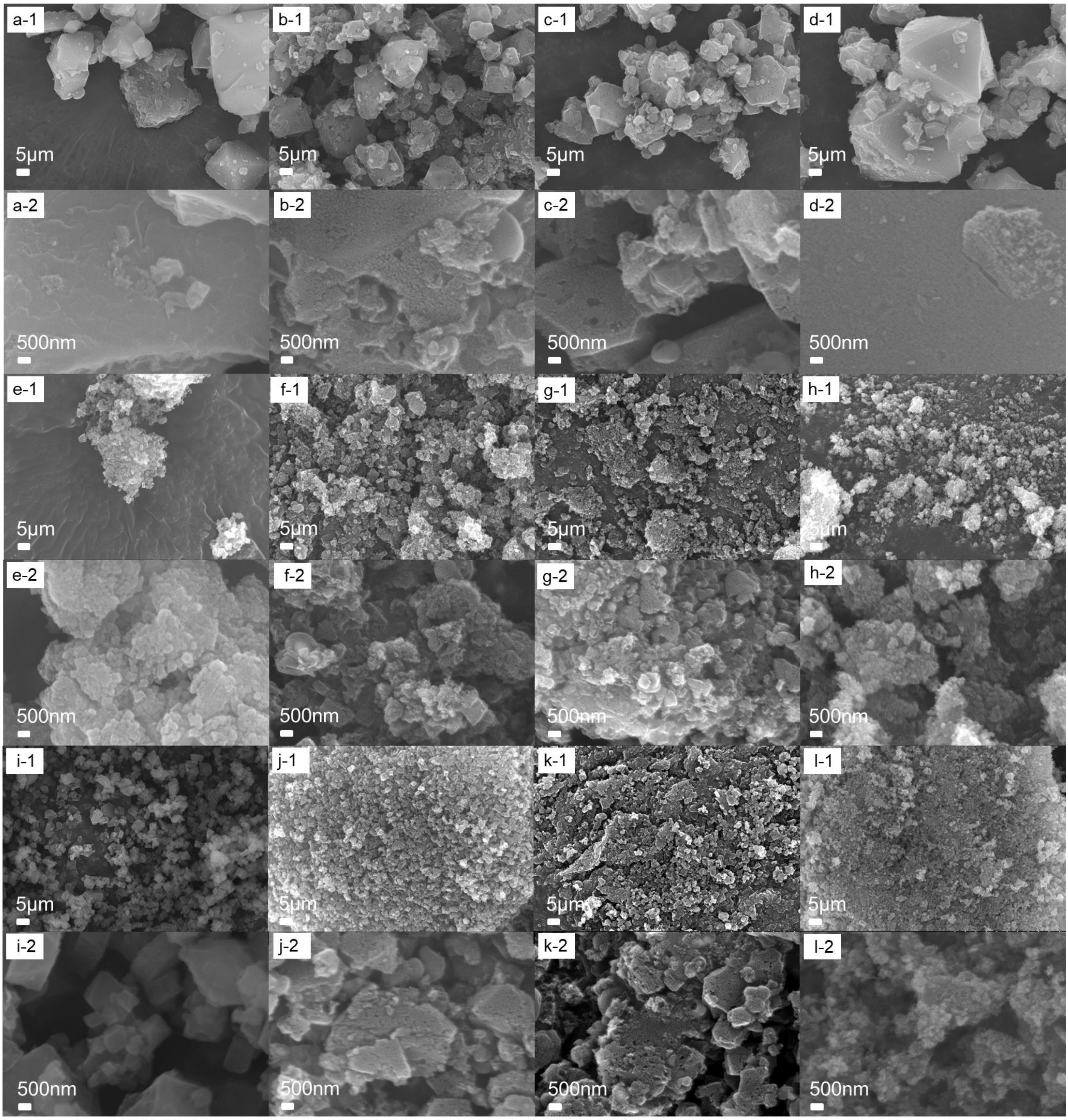

Figure S4. SEM images of low (-1) and high magnification (-2): (a) MIL-100 (Fe) iron; (b) MDC-A iron; (c) MDC-B iron; (d) MDMO iron; (e) MIL-100(Fe) nitrate; (f) MDC-A nitrate; (g) MDC-B nitrate; (h) MDMO nitrate; (i) MIL-100 (Fe) chloride; (j) MDC-A chloride; (k) MDC-B chloride; (l) MDMO chloride.

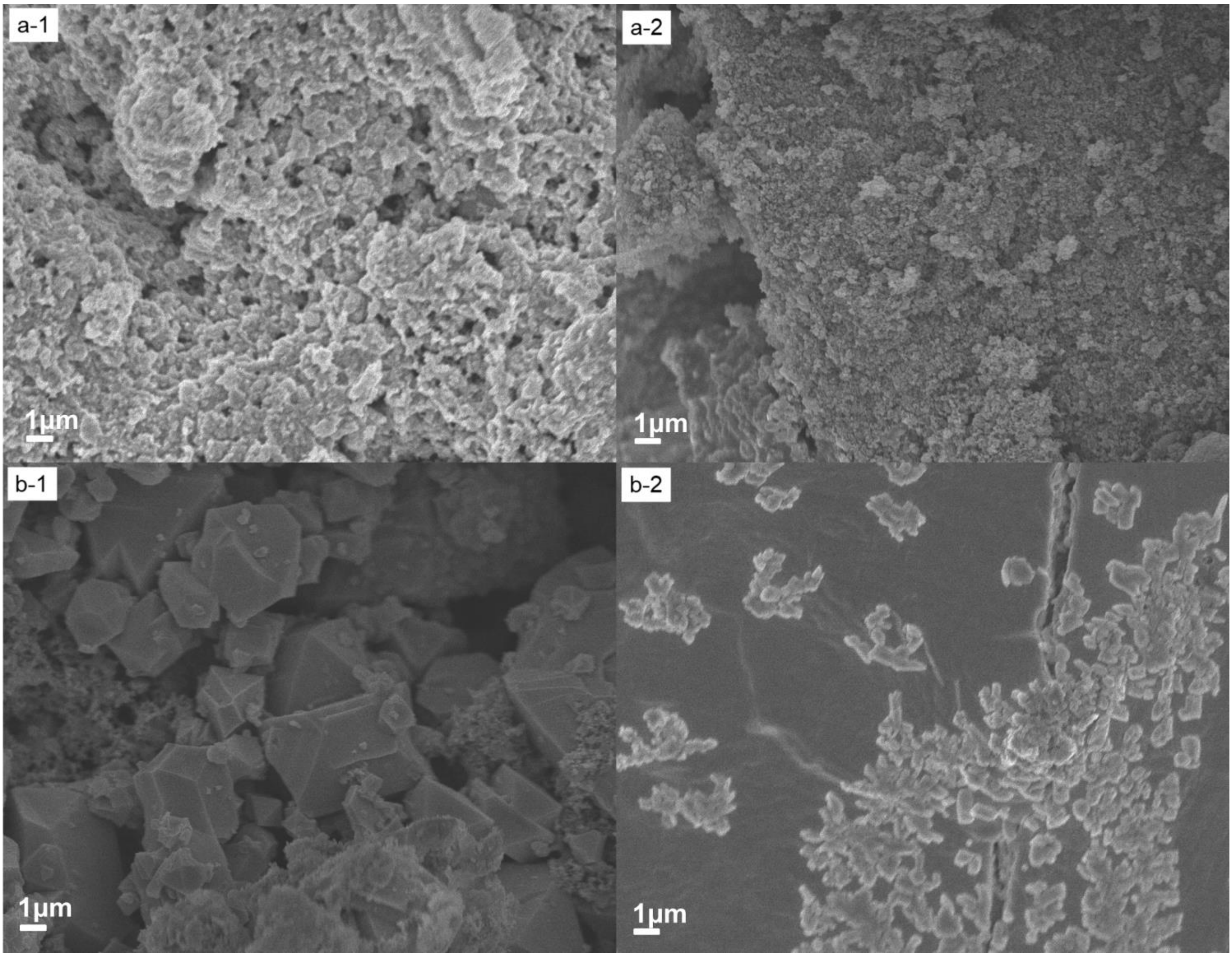

Figure S5. SEM images of before cycling test (-1) and after cycling test (-2): (a) MDC-A nitrate; (b) MDMO iron.

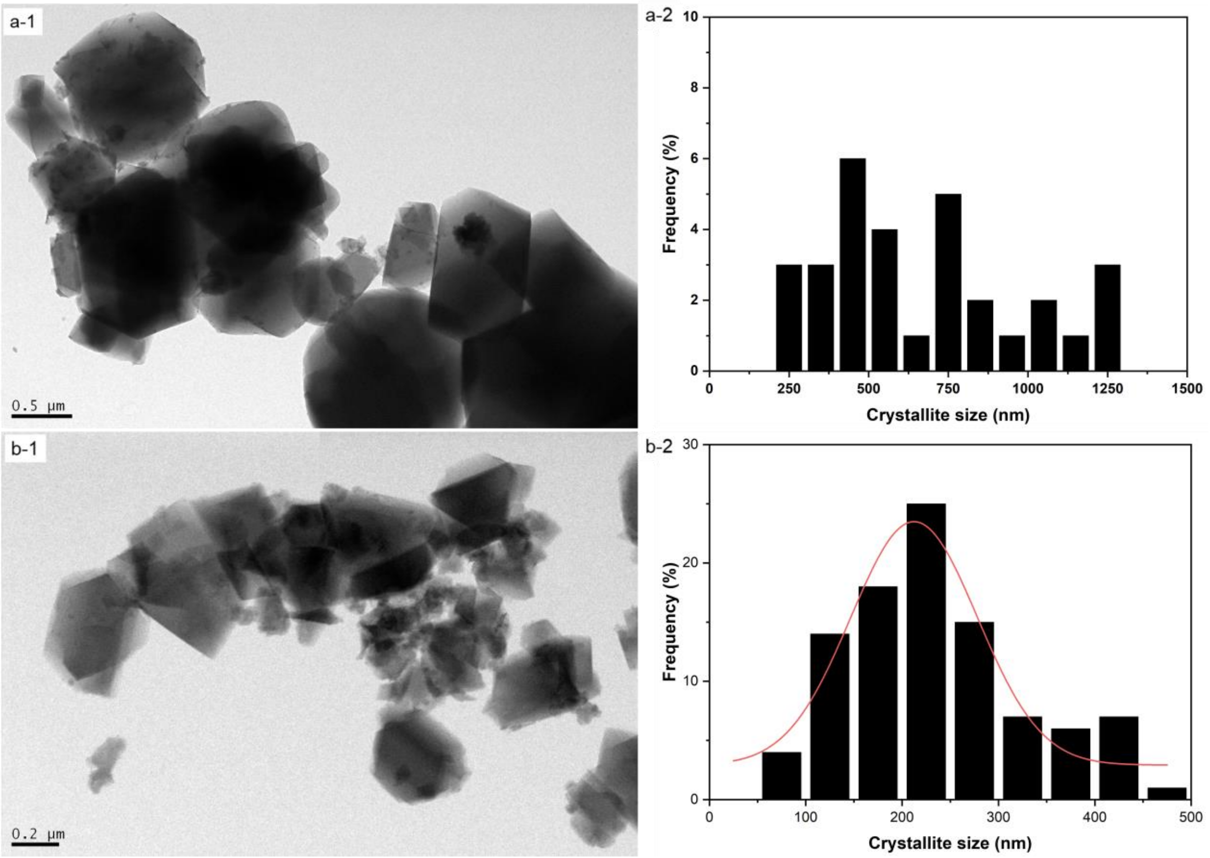

Figure S6. TEM images (-1) and crystallite size distribution (-2): (a) MIL-100 (Fe) iron; (b) MIL-100 (Fe) chloride.

Disclaimer/Publisher's Note: The statements, opinions and data contained in all publications are solely those of the individual author(s) and contributor(s) and not of MDPI and/or the editor(s). MDPI and/or the editor(s) disclaim responsibility for any injury to people or property resulting from any ideas, methods, instructions or products referred to in the content.
